# Effects of Rhodiola on production, health and gut development of broilers reared at high altitude in Tibet

**DOI:** 10.1038/srep07166

**Published:** 2014-11-24

**Authors:** Long Li, Honghui Wang, Xin Zhao

**Affiliations:** 1 College of Animal Science and Technology, Northwest A&F University, Yangling, Shaanxi, People's Republic of China; 2 College of Animal Science, Agricultural and Animal Husbandry College of Tibet University, Nyingchi, Tibet, People's Republic of China; 3 Department of Animal Science, McGill University, 21, 111 Lakeshore, Ste. Anne de Bellevue, Quebec, Canada, H9X 3V9

## Abstract

Rhodiola has long been used as a traditional medicine to increase resistance to physical stress in humans in Tibet. The current study was designed to investigate whether Rhodiola crenulata (R. crenulata) could alleviate the negative effects of hypoxia on broiler chickens reared in Tibet Plateau. The effect of supplementing crushed roots of R. crenulata on production performance, health and intestinal morphology in commercial male broilers was investigated. Dietary treatments included CTL (basal diet), Low-R (basal diet + 0.5% R. crenulata) and High-R (basal diet + 1.5% R. crenulata). In comparison with broilers fed the control diet, Low-R had no effect on production performance while High-R significantly decreased average daily feed intake at d14, 28 and 42, body weight at d28 and 42 and gut development. Ascites induced mortality did not differ among treatments. Nevertheless Low-R significantly reduced non-ascites induced mortality and total mortality compared with broilers fed CTL and High-R diets. Broilers fed the High-R diet had significantly increased blood red blood cell counts and hemoglobin levels at 28d compared with other treatments. Our results suggest that supplementation with Rhodiola might reduce the effects of hypoxia on broilers and consequently decrease mortality rate.

Poultry production in Tibet, China is increasing rapidly. It is well known that indigenous Tibet chickens are well adapted to high altitude environments and they mainly inhabit the areas around the midstream of Brahmaputra and Sanjiang rivers (about 2200 to 4000 m above sea level)^1^. Nevertheless, it has been a common practice for most poultry farmers in Tibet to raise commercial broiler chickens taking advantage of their fast growth rates acquired following intensive selection for this trait. However, these chickens are not well adapted to hypoxia, a main ecological factor with a negative impact on animal welfare and a threat to the survival of organisms in high altitude areas. Hypoxia refers to a low partial pressure of O_2_ in the inspired air. The partial pressure of O_2_ decreases with increasing altitude. Reduced levels of inspired O_2_ triggered acute pulmonary vasoconstriction and pulmonary hypertension in broilers^2^. Broilers raised in hypoxic environments exhibited lower growth performance^3^,^4^, altered gut development^5^ and blood parameters^6^, increased pulmonary arterial pressure and right ventricular hypertrophy^7^,^8^, and more importantly, increased mortality rates, especially ascites induced mortality^3^,^4^. Cost-effective measures are needed to ameliorate hypoxia induced negative effects on broiler production in Tibet and other high altitude regions.

Ascites is a cardiovascular metabolic disorder characterized by fluid accumulation in the abdominal cavity^4^. An estimated 4.7% of broilers worldwide have ascites, which causes great economic losses to the broiler industry^9^. The high incidence of ascites syndrome in modern broiler lines can be attributed to three major reasons as summarized by Kalmar et al^10^. Firstly, a superior growth rate inherently implies a high oxygen demand to sustain metabolic needs; secondly, the increased growth rate of modern broilers results in a significant reduction in relative heart and lung size, and thus diminished cardiopulmonary capacity; and thirdly, an increase in metabolic rates not only augments oxygen requirement at the tissue level, but also elevates mitochondrial production of reactive oxygen species^10^. The rate of ascites incidence could be much higher when commercial broiler strains are reared at high altitudes. Much effort has been made to prevent ascites syndrome in boiler chickens. Genetic selection for ascites resistant broiler lines could be a permanent solution. However, no such broiler lines have been successfully developed. Thus, feed restriction and changing lightering regimes have become the most common commercial conducts employed to reduce the incidence of ascites in broilers^4^. Both treatments slow down early growth and, unfortunately, they may not produce the same effects in broilers at high altitude. When birds were grown in a hypobaric chamber, ascites incidence increased while the overall growth rate of the birds was decreased^3^. Thus, alternative measures need to be developed to deal with ascites in broilers at high altitude.

A fast adaptation to high altitude conditions by broiler chickens is required to deal with hypoxia. This adaptation could be facilitated by adaptogens, which have to meet certain criteria proposed by Brekhman and Dardymov (1969)^11^. Rhodiola, a genus of perennial herbaceous plants in the family Crassulaceae, have been proposed as an adaptogen^12^. Rhodiola, known as “arctic root” or “golden root”, is able to increase resistance to a variety of stressors^12^. They have long been used in the treatment of long-term illness and weakness in people in Tibet for over 1000 years. Recent pharmacological studies have shown that Rhodiola possesses many health benefits such as ability to reduce fatigue^13^, is cytoprotective^14^, is anti-oxidative and can mitigate free radicals^14^,^15^, has immunomodulating properties^16^,^17^, is anti-hypoxia^18^ and can prevent altitude sickness^12^. Nevertheless, Rhodiola as an adaptogen has not been used in broilers raised in a hypoxic environment. Therefore, the objective of this research was to determine the effects of Rhodiola roots on production performance and health parameters of broilers raised at high altitude.

## Results

### Bird Performance

There were no significant differences in BW (body weight) at d14 among the three treatment groups. However, the BW of broilers fed the High-R diet (basal diet + 1.5% R. crenulata) was significantly lower at d28 and 42 (*p* = 0.003 and 0.019, respectively) as compared to birds fed the Low-R (basal diet + 0.5% R. crenulata) and CTL (basal diet) diets (Table 1). In comparison to the CTL diet, High-R diet significantly decreased ADFI (average daily feed intake) at all three 2-week periods (*p* = 0.047 at d14, 0.044 at d28 and 0.036 at d42). In comparison with the Low-R diet group, the High-R diet significantly decreased ADFI during the first two 2-week periods (*p* = 0.047). However, there were no significant differences in BW, ADFI and FCR (feed conversion ratio) between the CTL and the Low-R groups. The High-R treatment significantly reduced total feed intake during the six week period as compared with the other two treatments (*p* = 0.010). There were no significant differences in FCR among all three groups throughout the study.

### Mortality

**Figure 1:**
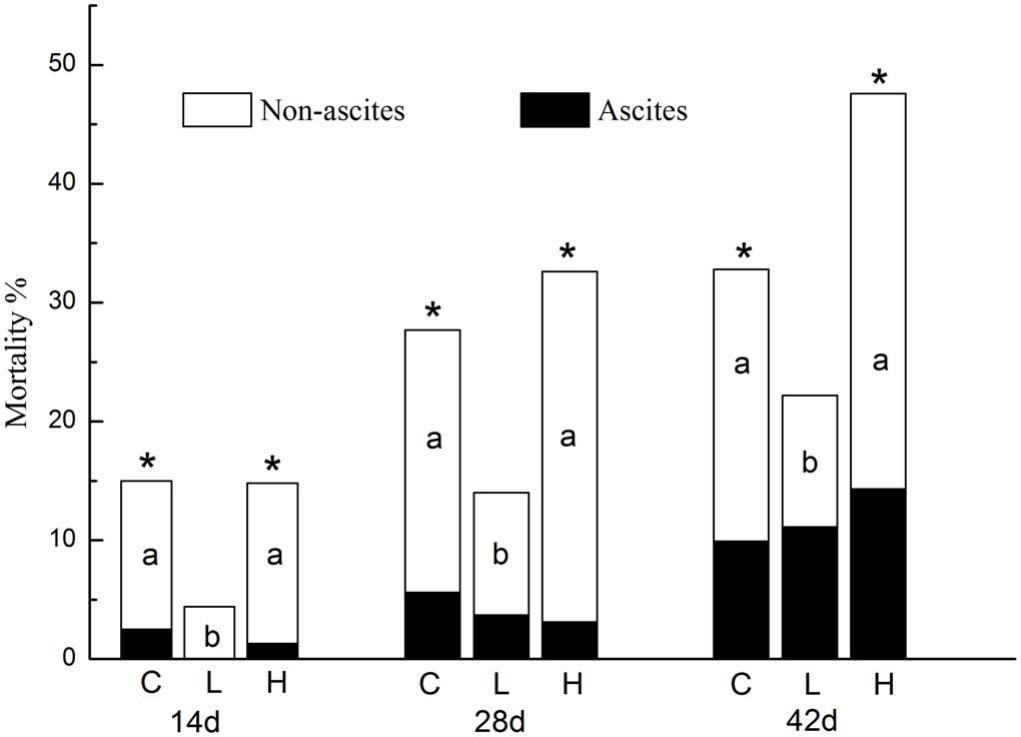
Effects of Rhodiola on mortality (ascites induced or non-ascites induced) of broiler chickens at high altitude. C: basal diet; L: CTL + 0.5% R.crenulata; H: CTL + 1.5% R.crenulata. * indicate the significant difference of the total mortality in comparison with the control group, while different litters (^a,b^) mean significant differences in non-ascites induced mortality in comparison with the control group.

As shown in Figure 1, there were no significant differences in ascites induced mortality among the three groups at all time periods. In addition, there were no significant differences of non-ascites induced mortality and total mortality between the CTL and the High-R groups. On the other hand, the Low-R diet significantly decreased non-ascites induced mortality and total mortality during all three 2-week periods (*p* = 0.006) (Figure 1).

### Organ index and blood parameters

There were no significant differences among the RV/TV (right ventricle weight/total ventricle weight) ratios of all three groups. Similarly, there was no change in TV/BW (total ventricle weight/body weight), except on 28 d, between the High-R and the Low-R groups. Compared to CTL, Low-R diet significantly decreased THW/BW (total heart weight/body weight) at d42 (*p* = 0.043). At the same time and as compared with the Low-R diet, the High-R diet significantly increased THW/BW (*p* = 0.043). While there was no significant difference in the LW/BW ratio on d14, the High-R group had a significantly higher LW/BW (lung weight/body weight) ratio than the control on d28 and d42 (*p* = 0.035 and 0.004, respectively) (Table 2).

As shown in Table 2, the High-R diet significantly increased blood RBC (red blood cell) counts, HGB (hemoglobin) levels and HGT (hematocrit) values on d28 in comparison with CTL and Low-R diets (*p* = 0.001, 0.001 and 0.002, respectively). In addition, the HGT value in Low-R group was significantly higher than values in the CTL group on d14 and d28 (*p* = 0.033 and 0.002, respectively). There were no significant differences in RBC counts, HGB levels and HGT values among all groups on d42.

### Histological Parameters

As shown in Table 3, the High-R diet significantly decreased the height of duodenum villi on d28 and d42 (*p* = 0.003 and 0.001, respectively) and jejunum villi on d42 (*p* = 0.029) when compared with the CTL and Low-R diets. At the same time, the High-R diet significantly decreased the height of ileum villi on d28 when compared with the Low-R diet (*p* = 0.021).

The effect of the three diets on crypt depth is presented in Table 4. In comparison with the CTL diet, the High-R diet significantly reduced duodenum crypt depth on d28 and d42 (*p* = 0.001 and 0.037, respectively), jejunum crypt depth on d14, d28 and d42 (*p* = 0.046, 0.044 and 0.031, respectively) and ileum crypt depth on d28 only (*p* = 0.044). The Low-R diet did not have any significant effects on crypt depth when compared with the CTL diet at all periods. The significant difference of crypt depth between the Low-R and the High-R diets was also observed for duodenum crypt depth on d42 (*p* = 0.001), for jejunum crypt depth on d28 and d42 (*p* = 0.044 and 0.031, respectively) and for ileum crypt depth on d28 (*p* = 0.044).

## Discussion

Our results support the notion that high altitude may reduce body weight. The final bodyweight of broilers in the CTL group at 2986m above sea level was 1347 g. This is approximately 35.5% lower than the expected growth performance of 2088 g at 42 d based on NRC (1994)^22^, despite the fact that broilers were fed a diet containing nutrients at NRC^22^ recommended levels. Our result is also in general agreement with the report of Balog et al (2000)^3^, who found that the final bodyweight of broilers raised in a hypobaric chamber (simulated 2900 m above sea level) was approximately 1650 g. The observed reduced body weight at high altitude could be due to a reduction in nutritional energy intake, a reduction in intestinal energy uptake as a result of impaired intestinal function and increased energy expenditure^23^. The ADFI was decreased by 30.8% in our control group as compared to NRC expected feed intake rate (NRC, 1994)^22^. Similarly, Westerterp et al. (2006)^24^ suggested that energy intake is the dominant determinant of body weight loss for humans under hypoxic conditions at high altitude. In addition, villi height and crypt depth were reduced in our study as compared with chickens raised at low altitude^21^, suggesting that absorption of nutrients at high altitude could be compromised. This observation is also supported by the finding of Santos et al (2005)^5^, who reported that simulated high altitude decreased gut development of broilers. Compromised intestinal development could be associated with decreased efficiency of nutrient absorption^25^. In comparison with the control group, the low level of R. crenulata had no effect on production parameters (BW, ADG, ADFI, FCR), but the high level of R. crenulata decreased the production performance of broilers, presumably due to the reduced feed intake caused by the poor taste of the feed.

The survival rate of lowland broilers appears to reduce when they are reared at high altitude such as in Tibet. Lowland chickens are susceptible to pulmonary hypertension due to the inadvertent consequence of selective breeding for rapid body growth and better feed conversion^26^. For many years, ascites has been a major cause of illness and death in meat-type chickens reared at high altitude (above 3,500 m)^7^. At high altitude, hypoxia forces the heart to greater activities due to pulmonary blood vessel constriction, which may result in a higher cardiac output, right ventricular hypertrophy and ascites^7^. Therefore, mortality in this study was categorized into ascites-induced and non-ascites-induced death. In addition to clinical symptoms such as serous fluid accumulation in the abdominal cavity^4^, hematological and anatomical changes were used to detect ascites^27^,^28^. An increase in the RV/TV ratio indicates the onset of pulmonary hypertension and ascites syndrome^29^,^30^,^31^,^32^. Hematocrit is another common measure of ascites development. Over 10% of ascites induced mortality in this study and the results of Özkan et al. (2010)^4^ clearly show that the hypobaric birds exhibited a significantly greater incidence of ascites. Based on our results, R. crenulata did not have anti-ascites potential under the conditions of this study.

The surprising and inspiring finding from this study was that the Low-R diet significantly reduced non-ascites induced mortality. However, whether the reduction was at least partially caused by higher HGT values on day14 and d28 is not clear. The High-R diet significantly increased blood RBC counts, HGB levels and HGT values on d28 in comparison with CTL and Low-R diets. However, the high level of R. crenulata affected the production parameters but did not improve the mortality rate. This could be caused by reduced feed intake and mal-absorption of nutrients indicated by compromised gut development. R. crenulata has a special smell that affects the palatability of feed when large amounts are added. Extracts are being considered for future research, on the condition that it has to be cost-effective. The components of R. crenulata that are responsible for our findings are elusive. Yang et al. (2012)^33^ reported the isolation of more than 100 compounds from Rhodiola, including phenols and their corresponding glycosides, cyanophoric glycosides, terpenoids, and flavonoids. Most studies for pharmacological functions of Rhodiola have used either whole parts of Rhodiola^34^ or extracts of Rhodiola^17^,^18^,^35^. Only a few studies have used purified components to test the pharmacological functions of R. crenulata in mostly in vitro experiments. For example, Chen et al (2012)^36^ found that four water-soluble phenylpropanoid compounds obtained from R. crenulata (p-hydroxyphenacyl-β-D-glucopyranoside, salidroside 2-(4-hydroxyphenyl)-ethyl-O-β-D-glucopyranosyl-6-O-β-D-glucopyranoside, and tyrosol) exhibited radical scavenging activities against 2,2-diphenyl-1-picrylhydrazy (DPPH) in vitro. Lee et al. (2013)^37^ found that R. crenulata extract and its bioactive components, salidroside and tyrosol, could maintain sodium transport via the preservation of Na, K-ATPase under hypoxia environment in vitro. Purified epicatechin-(4β, 8)-epicatechin gallate (B2-3′-O-gallate), epicatechin gallate (ECG) and 2-(4-hydroxyphenyl) ethyl 3,4,5-trihydroxybenzoate (HETB) from R. crenulata showed a strong α-glucosidase-inhibitory effect^38^. Similarly, both 4′-hydroxyacetophenone and epicatechin-(4β,8)-epicatechin gallate (B2−3′-O-gallate) inhibited xanthine oxidase (XO) activity, while salidroside and p-tyrosol did not show significant inhibitory effects on XO at 30 μM^39^. The R. crenulata components responsible for mitigating hypoxia induced pulmonary arterial pressure in this study are not clear. Therefore, more in-depth research must be carried out to elucidate key bioactive components and uncover definite mechanisms responsible for the adaptogenic potential of high altitude raised broilers observed in the present study. Although more research needs to be carried out before R. crenulata can be used in poultry production in Tibet, our findings lay a significant foundation for future studies.

## Methods

### Bird Husbandry

All experimental protocols used in this experiment were in accordance with those approved by the Northwest Agriculture and Forestry (A&F) University Institutional Animal Care and Use Committee (protocol number NWAFAC1008). A previous study has shown that ascites associated mortality in male broilers was higher (13.8%) than in female broilers (8.6%) under high altitude^4^. So to better create an ascites model that avoids sex difference in ascites associated mortality, hatched 1-d-old healthy Arbor Acres male broilers (n = 270) were obtained from a commercial hatchery at Chengdu (average altitude 500 m above sea level) and transported to the experimental farm of Tibet Agricultural and Animal Husbandry College (average altitude 2986 m above sea level) by air. Chicks were randomly allocated by body weight to 3 treatments (15 birds per cage with 6 cages per treatment) according to a completely randomized design. All chicks had similar initial weights (45.9 ± 0.8 g). Birds were reared in three layer metal cages, with an average stocking density of 16.7 birds per square meter from d1 to d14. Bird densities from d14 to d42 were 8.3 birds per square meter. The brooding temperature was 31 to 33°C during the first week and gradually decreased to 23°C by d21 until the end of the experiment. Daily fluorescent illumination was applied for 23 h with 1 h of dark throughout the experimental period of 42 d.

### Experimental Diets

Diets, in crumble form, included a starter diet (12.6 MJ metabolizable energy/kg of diet, 220 g/kg crude protein) from d1 to d21 and a grower diet (13 MJ metabolizable energy/kg of diet, 200 g/kg crude protein) from d22 to d42. The nutrient contents of the diets used in this study met or exceeded the National Research Council (1994) requirements. The control birds were fed the basal diet alone while birds on other treatments received the basal diet supplemented with 0.5% (Low-R diet) or 1.5% (High-R diet) Rhodiola crenulata (R. crenulata). There are 32 Rhodiola species in Tibet. Among these species, R. crenulata is widely used in Tibet to prevent high altitude sickness in humans. In addition, R. crenulata has been successfully cultivated by a botanist of Tibet Agricultural and Animal Husbandry College and the major bioactive components such as salidroside, tyrosol and flavonoid compounds are similar to those of wild R. crenulata (data not published). For this experiment, R. crenulata was purchased from the Tibetan Traditional Medical Hospital and was characterized by the botanist in the college. The roots of R. crenulata were used in this study because they are rich in biologically active compounds such as salidroside and tyrosol^40^,^41^,^42^. Dried roots of R. crenulata were crushed using a laboratory blender before mixing into the feed. The concentrations of major effective constituents in R. Crenulata (flavonoids, phenylpropanoids, and organic acids) measured by high-performance liquid chromatography were 7.55 mg/g for salidroside, 3.72 mg/g for tyrosol, 1.93 mg/g for gallic acid, 1.77 mg/g for rhodionin and 1.01 mg/g for rhodiosin. Feed and water were available at all times.

### Production Performance and Health Parameters

Birds were group-weighed at d14, d28 and d42 by cage and average daily gain (ADG) was calculated. Feed intake (FI) was determined weekly and feed consumption (FC) per bird (g/bird) was calculated by dividing the total FC of each cage by the actual number of birds in that cage. The feed conversion ratio (FCR) was determined as the FC per BW gain (g/g) for each cage in each period. Mortality was recorded daily. All dead birds during the experimental period were necropsied to identify ascites related mortality by characteristic symptoms, such as the presence of ascites fluid in the abdominal cavity, right ventricular dilation (RV/TV > 0.29), hydropericardium, and vascular congestion^19^. When none of the symptoms above occurred in a dead bird, the death was categorized as a non-ascites induced death. At d14, 28 and 42, two birds per cage (n = 12/treatment) were randomly selected and individually weighed. They were killed by cervical dislocation after taking blood samples from the heart. Hearts and lungs were thereafter removed and weighed to the nearest 0.1 g. The heart was resected and the atria were removed to the plane of the atrial ventricular valves followed by weighing the total ventricles (TV). The right ventricular (RV) wall was weighed after it was dissected free of the left ventricle (LV) and septum. Heart and lung weight relative to BW (g/100 g of BW) and the RV/TV ratio were calculated^20^.

Blood samples from all birds were analyzed for red blood cell (RBC) counts, hemoglobin (HGB) levels and hematocrit (HGT) values. These parameters were determined by an automatic blood analyzer (XFA6000, Pulang Company, Nanjing, China) that had been standardized for analyses of chicken blood.

### Gut Morphology

At 14, 28 and 42 d of age, 2 birds per treatment cage (n = 12/treatment) were randomly selected and sacrificed. Segments (1 cm) of duodenum (2 cm from gizzard), jejunum (adjacent to Meckel's diverticulum), and ileum (adjacent to cecal tonsils) were dissected, washed in physiological saline solution, and fixed in 10% buffered formalin^21^. For each intestinal segment, a 6-μm section was placed onto a glass slide and stained with hematoxylin and eosin for evaluation of villi height and crypt depth.

### Statistical analyses

Data were analyzed by the SPSS 13.0 software for windows (SPSS, Chicago, IL). Comparisons between groups were performed using one-way ANOVA followed by the Duncan test. For mortality, a χ2 analysis was performed for each group. Differences are considered statistically significant at the level of *p* < 0.05 and data are presented as means ± S.D.

## Author Contributions

L.L. and X.Z. contributed to the initial design of this experiment. L.L. and W.H. performed the experiment. L.L. analyzed the data. L.L. and X.Z. prepared the manuscript of this publication.

## Figures and Tables

**Table 1:** Effects of Rhodiola on production performance of broiler chickens at high altitude

	1–14 d			15–28 d			29–42 d			1–42 d	
Treatment^1^	BW(d14), g	ADFI, g	FCR	BW(d28), g	ADFI, g	FCR	BW(d42), g	ADFI, g	FCR	Total FI, g	FCR
CTL	172.6 ± 7.5	19.9 ± 0.46^a^	2.21 ± 0.08	514.5 ± 12.9^a^	49.4 ± 1.2^a^	2.03 ± 0.06	1347.3 ± 76.7^a^	114.6 ± 7.7^a^	1.93 ± 0.06	2574 ± 118^a^	1.98 ± 0.03
Low-R	173.0 ± 12.7	19.4 ± 0.44^ab^	2.15 ± 0.17	553.2 ± 38.2^a^	54.6 ± 4.3^a^	2.01 ± 0.04	1368.5 ± 114.2^a^	111.4 ± 13.9^ab^	1.92 ± 0.06	2595 ± 178^a^	1.96 ± 0.05
High-R	160.7 ± 11.6	18.1 ± 0.75^b^	2.21 ± 0.14	459.0 ± 53.6^b^	44.1 ± 5.9^b^	2.08 ± 0.06	1172.3 ± 144.8^b^	98.5 ± 13.7^b^	1.94 ± 0.03	2249 ± 249^b^	2.00 ± 0.04

^1^CTL: basal diet; Low-R: CTL + 0.5% R.crenulata; High-R: CTL + 1.5% R.crenulata.

^a, b^ Values with different superscripts within the same column are significantly different (*p* < 0.05).

BW, body weight; ADG, average daily gain; ADFI, average daily feed intake; FCR, feed conversion ratio; FI, feed intake.

**Table 2:** Effects of Rhodiola on cardiac and lung indices and blood parameters of broiler chickens at high altitude

	d14			d28			d42		
Item^1^	CTL	Low-R	High-R	CTL	Low-R	High-R	CTL	Low-R	High-R
RV/TV	0.24 ± 0.03	0.22 ± 0.02	0.23 ± 0.02	0.26 ± 0.02	0.25 ± 0.04	0.23 ± 0.03	0.26 ± 0.04	0.27 ± 0.04	0.27 ± 0.05
TV/BW	0.55 ± 0.08	0.48 ± 0.08	0.50 ± 0.08	0.49 ± 0.03^ab^	0.46 ± 0.04^b^	0.51 ± 0.04^a^	0.42 ± 0.05	0.39 ± 0.04	0.41 ± 0.04
THW/BW	0.61 ± 0.02	0.60 ± 0.02	0.61 ± 0.03	0.53 ± 0.04	0.49 ± 0.03	0.56 ± 0.03	0.46 ± 0.04^ab^	0.41 ± 0.02^c^	0.50 ± 0.04^a^
LW/BW	0.73 ± 0.03	0.71 ± 0.11	0.75 ± 0.07	0.69 ± 0.09^b^	0.78 ± 0.09^ab^	0.81 ± 0.09^a^	0.52 ± 0.04^b^	0.55 ± 0.06^ab^	0.62 ± 0.05^a^
RBC(10^12^/L)	2.40 ± 0.2	2.53 ± 0.13	2.57 ± 0.09	2.65 ± 0.21^a^	2.71 ± 0.19^a^	3.12 ± 0.17^b^	2.853 ± 0.36	3.08 ± 0.37	3.00 ± 0.41
HGB (g/L)	145.3 ± 10.6	152.0 ± 8.1	153.0 ± 8.2	153.5 ± 9.2^a^	160.0 ± 9.2^a^	179.5 ± 11.8^b^	170.5 ± 22.3	182.5 ± 24.8	176.8 ± 29.7
HGT (%)	27.3 ± 2.2^a^	29.7 ± 1.5^b^	28.5 ± 1.4^ab^	29.5 ± 2.4^a^	32.0 ± 2.1^b^	34.7 ± 1.4^c^	33.3 ± 3.9	35.8 ± 4.5	35.3 ± 5.6

CTL: basal diet; Low-R: CTL + 0.5% R.crenulata; High-R: CTL + 1.5% R.crenulata.

^a-c ^Values with different superscripts within the same row at the same time point are significantly different (*P* < 0.05).

^1^RV, right ventricles weight; TV, total ventricles weight; BW, body weight; LW, lung weight; THW, total heart weight; HGB, hemoglobin; HGT, hematocrit; RBC, red blood cell.

**Table 3:** Effects of Rhodiola on villi height of broiler chickens at high altitude μm

	Duodenum			Jejunum			Ileum		
Treatment^1^	d14	d28	d42	d14	d28	d42	d14	d28	d42
CTL	405.3 ± 19.6	514.4 ± 18.0^a^	709.9 ± 12.2^a^	268.7 ± 17.3	407.9 ± 15.1	429.1 ± 9.2^a^	172.3 ± 5.4	230.7 ± 15.2^ab^	335.5 ± 15.5
Low-R	407.3 ± 18.0	527.9 ± 9.8^a^	727.6 ± 17.8^a^	267.0 ± 23.0	411.2 ± 17.6	428.8 ± 13.9^a^	176.3 ± 6.4	235.7 ± 19.3^a^	338.2 ± 17.4
High-R	384.4 ± 14.2	497.1 ± 8.5^b^	684.4 ± 17.2^b^	254.0 ± 14.8	393.0 ± 13.0	411.9 ± 10.2^b^	169.3 ± 8.6	214.3 ± 13.0^b^	326.7 ± 11.4

^1^CTL: basal diet; Low-R: CTL + 0.5% R.crenulata; High-R: CTL + 1.5% R. crenulata.

^a, b^ Values with different superscripts within the same column are significantly different (*p* < 0.05).

**Table 4:** Effects of Rhodiola on crypt depth of broiler chickens at high altitude μm

	Duodenum			Jejunum			Ileum		
Treatment^1^	d14	d28	d42	d14	d28	d42	d14	d28	d42
CTL	101.5 ± 13.9	128.5 ± 5.7^a^	175.1 ± 5.2^a^	80.1 ± 6.0^a^	102.6 ± 5.9^a^	128.3 ± 7.8^a^	62.7 ± 2.4	78.3 ± 3.8^a^	103.0 ± 6.9
Low-R	107.9.3 ± 10.5	127.3 ± 8.2^ab^	181.1 ± 4.0^a^	79.1 ± 6.4^ab^	103.9 ± 5.4^a^	129.3 ± 5.6^a^	62.8 ± 4.5	77.6 ± 3.5^a^	108.7 ± 3.7
High-R	105.7 ± 13.4	120.8 ± 3.9^b^	167.5 ± 6.1^b^	72.9 ± 4.4^b^	95.5 ± 4.0^b^	118.2 ± 10.1^b^	62.2 ± 3.0	72.8 ± 3.7^b^	101.5 ± 6.5

^1^CTL: basal diet; Low-R: CTL + 0.5% R. crenulata; High-R: CTL + 1.5% R. crenulata.

^a, b^ Values with different superscripts within the same column are significantly different (*p* < 0.05).
